# Improved Nonlinear Extended State Observer-Based Sliding-Mode Rotary Control for the Rotation System of a Hydraulic Roofbolter

**DOI:** 10.3390/e24010041

**Published:** 2021-12-26

**Authors:** Zhen Zhang, Yinan Guo, Xianfang Song

**Affiliations:** 1 School of Information and Control Engineering, China University of Mining and Technology, Xuzhou 221116, China; zhenzhang@cumt.edu.cn; 2 School of Mechanical Electronic and Information Engineering, China University of Mining and Technology (Beijing), Beijing 100083, China

**Keywords:** extended state observer, sliding-mode surface, sliding-mode reaching law, sliding-mode control law, adaptation law

## Abstract

This paper develops a sliding-mode control with an improved nonlinear extended state observer (SMC-INESO) for the rotation system of a hydraulic roofbolter with dead-zones, uncertain gain, and disturbances, with the purpose of improving tracking performance. Firstly, the rotation system is modeled to compensate for dead-zone nonlinearity. Then, we present an improved nonlinear extended state observer to estimate disturbances of the rotation system in real time. Moreover, a proportional-integral-differential sliding-mode surface is introduced and an improved sliding-mode reaching law is designed. Based on this, a sliding-mode control law is developed. In order to eliminate the influence of estimation error and uncertain gain, we design two adaptation laws based on the sliding-mode surface and the estimated states. Finally, the effectiveness of the proposed SMC-INESO is verified through comparative simulation studies.

## 1. Introduction

**Figure 1:**
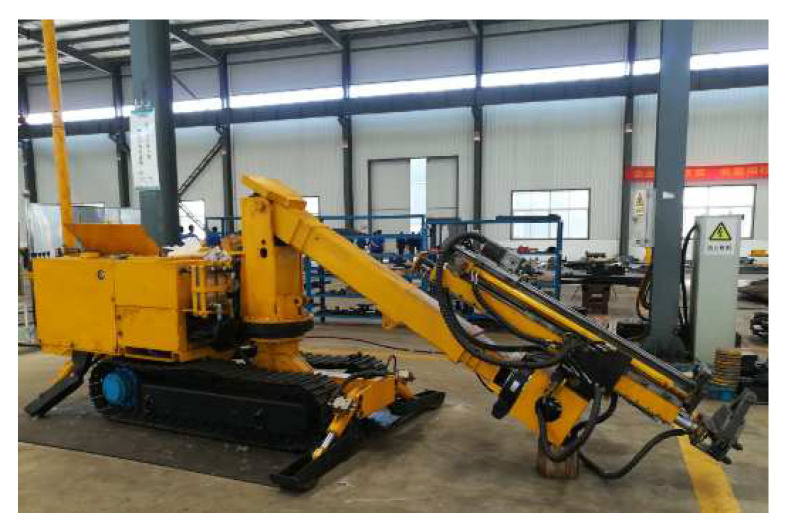
A hydraulic roofbolter.

A hydraulic roofbolter, as shown in Figure 1, is a key piece of mechanical equipment employed to install bolts in the roadway of a coal mine. The driller can effectively cut the rock and form holes by controlling the rotation system of a hydraulic roofbolter. After that, the bolts are installed in the holes to fix the surrounding rocks, thereby reliably supporting a roadway. This paper carries out rotary tracking control for the rotation system of a hydraulic roofbolter. High-performance rotary tracking control of the rotation system faces challenges due to dead-zones, disturbances (e.g., inherent disturbance and external disturbance), and uncertain gain [1,2]. In order to ensure the efficient and reliable drilling operation of the rotation system for a hydraulic roofbolter, its rotation speed must accurately track the preset value. Dead zones, disturbances, and other uncertain factors may deteriorate the tracking control of the rotation system. Inappropriate rotation speed of drill pipe may reduce drilling efficiency, even causing broken drill pipes [3]. Therefore, it is of great importance to achieve precise tracking control of the rotation system so as to ensure efficient and reliable roadway support.

In order to deal with the aforementioned negative factors and improve tracking performance, a large number of nonlinear control methods, such as backstepping control [3,4], sliding-mode control [5,6,7,8], and active disturbance rejection control [9,10], have been developed. Thanks to the simplicity and strong robustness of sliding-mode control, it has been widely used to cope with the uncertainty of the system [11,12]. Specifically, Rojsiraphisal et al. [11] developed a novel fast terminal sliding-mode control method based on a disturbance observer for a robot system. The disturbance observer can estimate the external disturbance of the system in a limited time, which enhances the anti-disturbance ability of a robot system. The control performance is improved by compensating for the estimated disturbance with the sliding-mode control law. Mobayen et al. [12] presented a novel adaptive intelligent global sliding-mode controller for tracking control of DC-DC buck converters subjected to dynamic uncertainties and disturbances. However, chattering in sliding-mode control has always been the main problem that limits its application and development. In existing sliding-mode control methods, controllers with robust switching of the sign function are used to suppress the disturbances [13,14,15]. Controllers with sign functions inevitably create system chattering. Therefore, improper disturbance processing is the main cause of chattering in sliding mode control.

To the best of our knowledge, control methods based on a disturbance observer can effectively enhance the anti-disturbance ability and improve the control performance of systems [16]. Based on this, in order to better suppress chattering, disturbance observer-based sliding-mode control has received extensive attention. As we know, extended state observer (ESO) as a mature disturbance estimation technology has superior estimation performance [17,18,19,20]. Therefore, ESO-based sliding-mode control has been widely used in many control fields [21,22,23,24,25,26]. Liu et al. [21] proposed an ESO-based SMC by combining a super switching approach and a linear ESO for power converters subjected to external load. The former was used to quickly track the preset signal, while the disturbance was estimated by a linear ESO and compensated for by the controller. Both guaranteed efficient control performance. To deal with the external and internal disturbances of a wind power system, Wang et al. [22] presented an adaptive SMC based on a linear ESO, which weakened sliding chattering and improved anti-disturbance ability. In order to cope with the dynamic disturbance and uncertainties of steer-by-wire systems, as well as time-varying road environments, Sun et al. [23] designed a nonlinear ESO-based SMC. The negative effects of uncertainties and the time-varying road environment on the system were suppressed by the SMC, thereby improving the robustness of the controller. After the dynamic disturbance was estimated by a nonlinear ESO, it was compensated for by a controller so as to weaken its influence on the system. Considering dynamic nonlinearity and external disturbance, Wang et al. [24] proposed a fuzzy sliding-mode control method by integrating active disturbance rejection control with fuzzy sliding-mode control. After estimating dynamic nonlinearities and external disturbances by a linear ESO, a fuzzy sliding-mode control law was designed based on their estimation errors. Wu et al. [25] proposed a terminal sliding-mode controller based on a linear ESO by combining sliding-mode control and active disturbance rejection control for the purpose of dealing with internal dynamic uncertainty and external disturbance in a vehicle system. The tracking performance was improved by compensating for the estimated uncertainty and disturbance in the designed nonlinear sliding-mode control law in terms of estimation errors. In order to solve the internal and external disturbances in a motor system, Alonge et al. [26] developed an active disturbance rejection controller based on a sliding-mode component. In this controller, two linear ESOs were employed to estimate internal and external disturbances. All estimated disturbances were compensated for by the sliding-mode component, which improved the robustness and the anti-disturbance ability of the systems. However, the aforementioned control methods still have the following two challenges that need to be resolved. One is that conventional linear or nonlinear ESOs with large gains are employed to estimate disturbances. Large gains ESOs may amplify noise, thereby reducing control performance. The other is that the estimation error of disturbance inevitably appears in the ESO, which may reduce the control performance. Previous studies usually employed robust switching with a sign function to suppress the estimation error, leading to chattering.

Along with the above-mentioned challenges, dead zone and uncertain gain of the rotation system for a hydraulic roofbolter also worsen control performance. In order to tackle these problems, this paper proposes a sliding-mode control with an improved nonlinear extended state observer (SMC-INESO). Firstly, the rotation system is modeled after compensating for the dead zone. Following that, an improved nonlinear extended state observer with smaller gain is developed to quickly estimate disturbances for the purpose of effectively estimating the disturbances and suppressing noise. Moreover, a sliding-mode control law is developed based on a proportional-integral-differential sliding-mode surface and an improved sliding-mode reaching law. In order to eliminate the influence of estimation error and uncertain gain, two adaptation laws are designed based on the sliding-mode surface and control signal. This paper has the following four contributions:

1. Proposing an improved nonlinear extended state observer with smaller gain, with the purpose of effectively estimating the disturbances of the rotation system and reducing the influence of noise.
2. Developing an improved sliding-mode reaching law to improve system robustness.
3. Presenting a sliding-mode control law, which aims to provide a continuous and effective control law for the rotation system.
4. Designing two adaptation laws to tune the estimations of disturbance estimation error and uncertain gain and then compensate for them in the sliding-mode control law.

The rest of the paper is organized as follows. Section 2 models the rotation system model after compensating for the dead zone nonlinearity; Section 3 proposes and analyzes the SMC-INESO for the rotation system; Section 4 carries out comparative simulations and analyzes the experiment results. Finally, the whole paper is concluded in Section 5.

## 2. Model of the Rotation System

**Figure 2:**
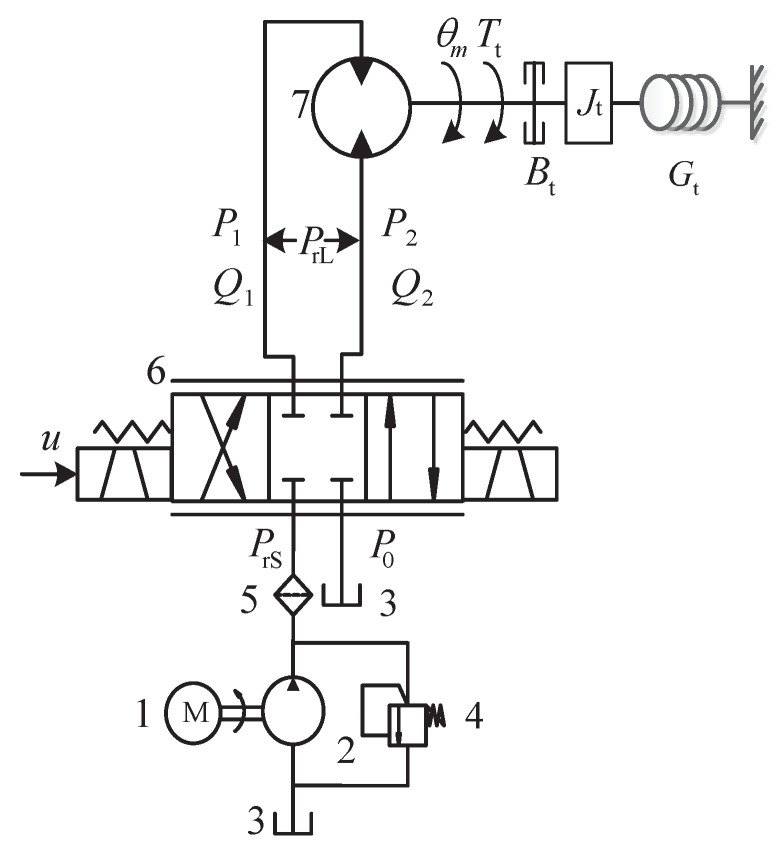
The schematic diagram of the rotation system (1—asynchronous motor, 2—quantitative pump, 3—tank, 4—relief valve, 5—filter, 6—hydraulic valve, 7—hydraulic motor).

The structure diagram of the rotation system for a hydraulic roofbolter is shown in Figure 2. In the rotation system, hydraulic oil is pumped by a quantitative pump driven by an asynchronous motor and the rotation speed is controlled by a hydraulic valve. The rotation speed changes with the flow of hydraulic oil and the flow is adjusted by the above-mentioned hydraulic valve in order to accurately track the preset rotation speed. Therefore, the hydraulic motor and hydraulic valve are the core actuators of the rotation system. This section builds a rotation system model based on the dynamics of the above components.

### 2.1. Dynamics of the Hydraulic Motor

The load dynamic of the hydraulic motor in the rotation system is presented as follows:

(1) $$D_{m}{\dot{\theta }}_{m}=Q_{\mathrm{rL}}-C_{\mathrm{tm}}P_{\mathrm{rL}}-\frac{V_{r}}{4\beta }{\dot{P}}_{\mathrm{rL}}+q\left(t\right)$$

where $D_{m}$ is the displacement of the hydraulic motor, $\theta_{m}$ is the rotary angle, ${\dot{\theta }}_{m}$ is the rotation speed, $Q_{\mathrm{rL}}$ is the load flow of the hydraulic motor, $C_{\mathrm{tm}}$ is the leakage coefficient, $P_{\mathrm{rL}}$ is the load pressure of the hydraulic motor, $V_{r}$ is the volume of the hydraulic motor chamber, β is the bulk modulus of hydraulic oil, and q(t) represents un-modeled disturbances, such as friction and external disturbances.

The torque dynamics of the hydraulic motor in the rotation system can be described as follows:

(2) $$D_{m}P_{\mathrm{rL}}=J_{t}{\ddot{\theta }}_{m}+B_{t}{\dot{\theta }}_{m}+G_{t}\theta_{m}+T_{t}+\Delta_{r}$$

where $T_{t}$ is the load torque of the hydraulic motor; $J_{t}$ is the total inertia; $B_{t}$ is the total equivalent viscous damping coefficient; $G_{t}$ is the total equivalent stiffness; $\Delta_{r}$ represents the modeling error.

### 2.2. Dynamics of the Hydraulic Valve

The dynamics of the load flow in the hydraulic valve is represented as follows:

(3) $$Q_{\mathrm{rL}}\;=\;C_{d}Wx_{v}\sqrt{\frac{P_{\mathrm{rs}}-\mathrm{sgn}\left(x_{v}\right)P_{\mathrm{rL}}}{\rho }}$$

where $C_{d}$ is the flow coefficient, *W* is the gradient area of the hydraulic valve, $x_{v}$ is the spool displacement of the hydraulic valve, ρ is the emulsion density of the hydraulic oil, and sgn(·) represents the following function.

(4) $$\mathrm{sgn}\left(\cdot \right)=\left\{\begin{array}{cc} 1 & \cdot >0\\ 0 & \cdot =0\\ -1 & \cdot <0 \end{array}\right.$$

**Figure 3:**
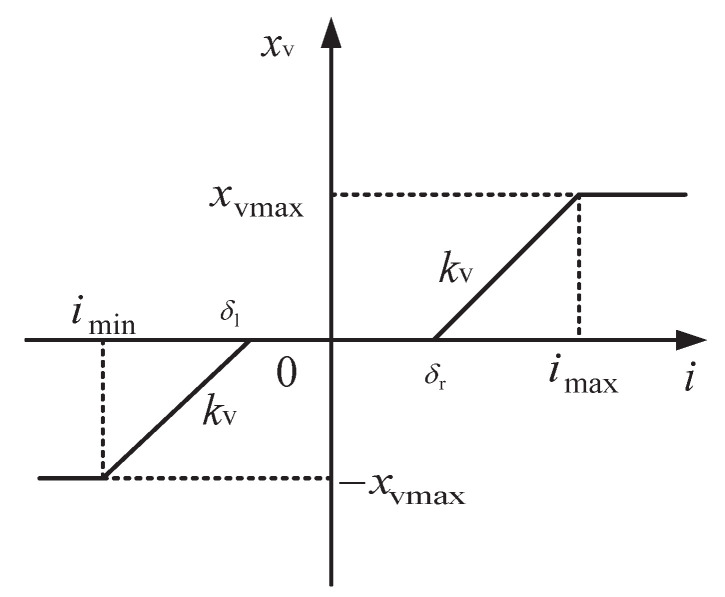
The dead zone of pool displacement in the hydraulic valve.

In the rotation system, the hydraulic valve is trapped in the dead zone nonlinearity [3], as shown in Figure 3. Then, the dynamic of spool displacement of the hydraulic valve is formulated as follows:

(5) $$x_{v}=\left\{\begin{array}{cc} -x_{\mathrm{vmax}} & i<i_{\min}\\ k_{v}\left[i-d\left(i\right)\right] & i_{\min}\leq i\leq i_{\max}\\ x_{\mathrm{vmax}} & i>i_{\max} \end{array}\right.$$

with

(6) $$d\left(i\right)=\left\{\begin{array}{cc} \delta_{l} & i\leq \delta_{l}\\ i & \delta_{l}<i\leq \delta_{r}\\ \delta_{r} & i\geq \delta_{r} \end{array}\right.$$

In the above Equations (5) and (6), $k_{v}$ is the proportional coefficient and $x_{\mathrm{vmax}}$ is the maximum pool displacement. $\delta_{r}$ and $\delta_{l}$ are the unknown dead zone boundary parameters. Define $\delta ={\left[\delta_{r},\delta_{l}\right]}^{T}$, and denote $\hat{\delta }={\left[{\hat{\delta }}_{r},{\hat{\delta }}_{l}\right]}^{T}$ and $\tilde{\delta }=\hat{\delta }-\delta$ as the estimation and estimation error vectors of δ.

By employing the dead zone compensation method proposed in [1], Equation (3) can be rewritten as follows:

(7) $$Q_{\mathrm{rL}}\;=\;C_{d}Wk_{v}\left[u+{\tilde{\delta }}^{T}\left(\bar{\alpha }+\bar{\beta }\right)\right]\sqrt{\frac{P_{\mathrm{rS}}-\mathrm{sgn}\left(x_{v}\right)P_{\mathrm{rL}}}{\rho }}$$

In the above formula, $\bar{\alpha }=[\alpha ,1-\alpha {]}^{T}$ and $\bar{\beta }={\left[\alpha \cdot sgn\left({\tilde{\delta }}_{r}\right),(\alpha -1)\cdot sgn\left({\tilde{\delta }}_{l}\right)\right]}^{T}{\mathrm{sat}}_{\tilde{\delta }}\left(u\right)$. α and ${\mathrm{sat}}_{\tilde{\delta }}\left(u\right)$ are represented as follows:

(8) $$\alpha =\left\{\begin{array}{cc} 0 & u<0\\ 1 & u\geq 0 \end{array}\right.$$

(9) $${\mathrm{sat}}_{\tilde{\delta }}\left(u\right)=\left\{\begin{array}{cc} 1+\frac{u}{{\tilde{\delta }}_{r}}, & 0\leq u<-{\tilde{\delta }}_{r}\;\mathrm{and}\;{\tilde{\delta }}_{r}<0,\\ 1+\frac{u}{{\tilde{\delta }}_{l}}, & -{\tilde{\delta }}_{l}<u<0\;\mathrm{and}\;{\tilde{\delta }}_{l}>0,\\ 0, & otherwise. \end{array}\right.$$

Based on Equations (1), (2), and (7), we obtain

(10) $$\begin{array}{cc} {\dddot{\theta }}_{m} & =-\frac{4\beta C_{\mathrm{tm}}G_{t}}{V_{r}J_{t}}\theta_{m}-\frac{4\beta D_{m}^{2}+4\beta C_{\mathrm{tm}}B_{t}+V_{r}G_{t}}{V_{r}J_{t}}{\dot{\theta }}_{m}\\ \; & -\frac{4\beta C_{\mathrm{tm}}J_{t}+V_{r}B_{t}}{V_{r}J_{t}}{\ddot{\theta }}_{m}\\ \; & +\frac{4\beta C_{d}Wk_{v}D_{m}}{V_{r}J_{t}\sqrt{\rho }}\sqrt{P_{\mathrm{rS}}-\mathrm{sgn}\left(x_{v}\right)P_{\mathrm{rL}}}u\\ \; & -\frac{4\beta C_{d}Wk_{v}D_{m}}{V_{r}J_{t}\sqrt{\rho }}\sqrt{P_{\mathrm{rS}}-\mathrm{sgn}\left(x_{v}\right)P_{\mathrm{rL}}}{\tilde{\delta }}^{T}\left(\bar{\alpha }-\bar{\beta }\right)\\ \; & -\frac{1}{V_{r}J_{t}}\left[4\beta C_{\mathrm{tm}}\left(T_{t}+\Delta_{r}\right)+V_{r}\left({\dot{T}}_{t}+{\dot{\Delta }}_{r}\right)-4\beta D_{m}q\left(t\right)\right] \end{array}$$

### 2.3. Formulation of the Rotation System

Denote ${\left[x_{1},x_{2},x_{3}\right]}^{T}={\left[\theta_{m},{\dot{\theta }}_{m},{\ddot{\theta }}_{m}\right]}^{T}$ as the state variables. *u* is the control input and *y* is the system output. Based on Equation (10), the rotation system is modeled as follows:

(11) $$\left\{\begin{array}{c} {\dot{x}}_{1}=x_{2}\\ {\dot{x}}_{2}=x_{3}\\ {\dot{x}}_{3}=f\left(t\right)u+\gamma \left(t\right)\\ y=x_{2} \end{array}\right.$$

In the above model, $f\left(t\right)=\frac{4\beta C_{d}Wk_{v}D_{m}}{V_{r}J_{t}\sqrt{\rho }}\sqrt{P_{\mathrm{rS}}-\mathrm{sgn}\left(x_{v}\right)P_{\mathrm{rL}}}$, and $\gamma \left(t\right)=-\frac{4\beta C_{\mathrm{tm}}G_{t}}{V_{r}J_{t}}x_{1}-\frac{4\beta D_{m}^{2}+4\beta C_{\mathrm{tm}}B_{t}+V_{r}G_{t}}{V_{r}J_{t}}x_{2}-\frac{4\beta C_{\mathrm{tm}}J_{t}+V_{r}B_{t}}{V_{r}J_{t}}x_{3}-\frac{4\beta C_{d}Wk_{v}D_{m}}{V_{r}J_{t}\sqrt{\rho }}\sqrt{P_{\mathrm{rS}}-\mathrm{sgn}\left(x_{v}\right)P_{\mathrm{rL}}}{\tilde{\delta }}^{T}\left(\bar{\alpha }-\bar{\beta }\right)-\frac{1}{V_{r}J_{t}}$$\left[4\beta C_{\mathrm{tm}}\left(T_{t}+\Delta_{r}\right)+V_{r}\left({\dot{T}}_{t}+{\dot{\Delta }}_{r}\right)-4\beta D_{m}q\left(t\right)\right]$ presents the “total disturbance” in the rotation system. Since the physical parameters, such as β, $C_{\mathrm{tm}}$ and $k_{v}$, are time-varying, f(t) is an uncertain gain.

## 3. Design and Analysis of the Proposed SMC-INESO

**Figure 4:**
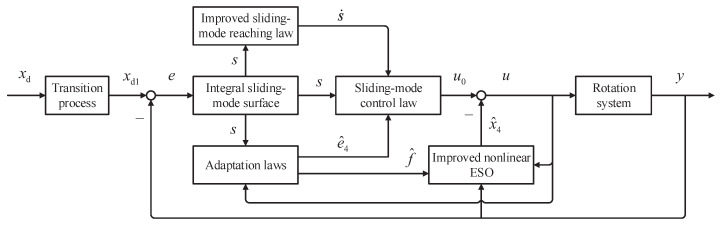
The framework of the proposed controller.

In this section, for the purpose of improving the tracking performance of the rotation system, a sliding-mode control with an improved nonlinear extended state observer (SMC-INESO) is proposed. As shown in Figure 4, the proposed SMC-INESO is composed of the transition process, the improved nonlinear ESO, the integral sliding-mode surface, the improved sliding-mode reaching law, the sliding-mode control law, and the adaptation laws.

### 3.1. The Transition Process

The rotation speed set according to rock properties, expressed by $x_{d}$, is a step signal. Overshoot may occur when the system quickly tracks the signal. To solve the issue, the following discrete second-order transition process is employed to convert the step signal to a continuous one [27].

(12) $$\left\{\begin{array}{c} {x_{d}}_{1}\left(k+1\right)={x_{d}}_{1}\left(k\right)+h\cdot {x_{d}}_{2}\left(k\right)\\ {x_{d}}_{2}\left(k+1\right)={x_{d}}_{2}\left(k\right)+h\cdot u_{1} \end{array}\right.$$

with

(13) $$\left\{\begin{array}{c} d=rh_{0},\;d_{0}=h_{0}d,\;h_{0}>h\\ y^{\prime }={x_{d}}_{1}-x_{d}+h_{0}{x_{d}}_{2},\;a_{0}=\sqrt{d^{2}+8r\left|y^{\prime }\right|}\\ \begin{array}{c} a_{1}=\left\{\begin{array}{cc} {x_{d}}_{2}+\frac{a_{0}-d}{2} & |y^{\prime }|>d_{0}\\ {x_{d}}_{2}+\frac{y^{\prime }}{h_{0}} & |y^{\prime }|\leq d_{0} \end{array}\right. \end{array}\\ \begin{array}{c} u_{1}=-\left\{\begin{array}{cc} r\cdot \mathrm{sgn}(a_{1}) & |a_{1}|>d\\ r\cdot \frac{a_{1}}{d} & |a_{1}|\leq d \end{array}\right. \end{array} \end{array}\right.$$

In the above formulas, ${x_{d}}_{1}$ is the output of the transition process as well as the tracked signal, $k\in N^{+}$; r>0; *h* is the integration step; $u_{1}$ is a fast control function proposed by Han [27], in which, *d*, $d_{0}$, and $h_{0}$ are determined by *r* and *h*.

### 3.2. The Improved Nonlinear Extended State Observer

Traditional linear or nonlinear ESOs are widely employed by existing control methods [21,22,23,24,25,26] to estimate disturbances. In order to obtain better estimation performance, decision makers usually provide large gains for ESOs. However, large gains of ESOs may amplify noise, thereby reducing control performance. In view of this, if the gains of ESOs are reduced under the premise of ensuring good estimation performance of ESOs, this will inevitably reduce the influence of noise and improve the control performance of a system. In order to achieve the above goals, we design the adaptive gains based on the estimated error of x2, and then develop an improved nonlinear ESO. Due to the suppression of noise, the proposed observer will contribute to good tracking performance and efficient operation of the rotation system in a roadway.

Denote $x_{4}=\gamma \left(x,t\right)-\tilde{f}u$ as the extended state and define ${\dot{x}}_{4}={\left(\gamma \left(x,t\right)-\tilde{f}u\right)}^{\prime }=\gamma_{1}\left(x,t\right)$. Then, the traditional extended state observer [28] is expressed as follows:

(14) $$\left\{\begin{array}{c} e_{2}={\hat{x}}_{2}-y\\ {\dot{\hat{x}}}_{2}={\hat{x}}_{3}-\beta_{01}\varphi_{1}\left(e_{2}\right)\\ {\dot{\hat{x}}}_{3}=\hat{f}u+{\hat{x}}_{4}-\beta_{02}\varphi_{2}\left(e_{2}\right)\\ {\dot{\hat{x}}}_{4}=-\beta_{03}\varphi_{3}\left(e_{2}\right)+\gamma_{1}(\hat{x},t) \end{array}\right.$$

In the above formula, $\hat{f}$ is the estimation of *f*, which is tuned by the adaptation law designed later. $\beta_{0j}>0$, j=1,2,3 are the gains. $\varphi_{j}\left(e_{2}\right)$ are expressed as follows:

(15) $$\varphi_{j}\left(e_{2}\right)=\mathrm{fal}(e_{2},\alpha_{j},\delta )=\left\{\begin{array}{cc} |e_{2}{|}^{\alpha_{j}}\mathrm{sgn}\left(e_{2}\right) & |e_{2}|>\delta \\ \frac{e_{2}}{\delta {}^{\left(1-\alpha_{j}\right)}} & |e_{2}|\leq \delta \end{array}\right.$$

where fal(·) is a function proposed by Han, $0<\alpha_{3}<\alpha_{2}<\alpha_{1}=1$, 0<δ<1; for more details about its parameter settings, please refer to [28].

Note that Equation (14) is a linearly extended state observer when $\alpha_{j}=1$. When $e_{2}$ tends to 0, $\varphi_{j}\left(e_{2}\right)$ tends to 0. In view of this, in order to ensure an effective estimation of the disturbance, $\beta_{0j}$ are often set to relatively large values. However, large gains will amplify noise, which will inevitably reduce control performance. To solve the above problem, this section designs an improved nonlinear extended state observer with smaller gains to effectively estimate the disturbance as follows:

(16) $$\left\{\begin{array}{c} e_{2}={\hat{x}}_{2}-y\\ {\dot{\hat{x}}}_{2}={\hat{x}}_{3}-\beta_{01}\lambda_{01}\left(e_{2}\right)e_{2}\\ {\dot{\hat{x}}}_{3}=\hat{f}u+{\hat{x}}_{4}-\beta_{02}\lambda_{02}\left(e_{2}\right)e_{2}\\ {\dot{\hat{x}}}_{4}=-\beta_{03}\lambda_{03}\left(e_{2}\right)e_{2}+\gamma_{1}(\hat{x},t) \end{array}\right.$$

with

(17) $$\lambda_{0j}\left(e_{2}\right)=e^{k_{j}{\left|e_{2}\right|}^{\alpha }}=\left\{\begin{array}{cc} e^{k_{j}{\left|e_{2}\right|}^{\alpha_{1}}} & |e_{2}|<1\\ e^{k_{j}{\left|e_{2}\right|}^{\alpha_{2}}} & |e_{2}|\geq 1 \end{array}\right.$$

In the above formulas, $\alpha_{1}<\alpha_{j}-1<\alpha_{2}$, $k_{1}\geq 1$, $k_{2}=2k_{1}$, $k_{3}=3k_{1}$. Note that, since $e^{k_{j}{\left|e_{2}\right|}^{\alpha_{1}}}=e^{k_{j}{\left|e_{2}\right|}^{\alpha_{2}}}=e^{k_{j}}$ as $|e_{2}|=1$, $\lambda_{0j}\left(e_{2}\right)$ is a continuous function.

The traditional extended state observer, represented by Equation (14), can be transformed as follows:

(18) $$\left\{\begin{array}{c} e_{2}={\hat{x}}_{2}-y\\ {\dot{\hat{x}}}_{2}={\hat{x}}_{3}-\beta_{01}\lambda_{1}\left(e_{2}\right)e_{2}\\ {\dot{\hat{x}}}_{3}=\hat{f}u+{\hat{x}}_{4}-\beta_{02}\lambda_{2}\left(e_{2}\right)e_{2}\\ {\dot{\hat{x}}}_{4}=-\beta_{03}\lambda_{3}\left(e_{2}\right)e_{2}+\gamma_{1}(\hat{x},t) \end{array}\right.$$

with

(19) $$\lambda_{j}\left(e_{2}\right)=\left\{\begin{array}{cc} |e_{2}{|}^{\alpha_{j}-1} & |e_{2}|>\delta \\ \delta {}^{\alpha_{j}-1} & |e_{2}|\leq \delta \end{array}\right.$$

Comparing Equations (16) and (18), we can obtain $e^{k_{j}{\left|e_{2}\right|}^{\alpha_{1}}}>k_{j}{\left|e_{2}\right|}^{\alpha_{1}}>\delta^{\alpha_{j}-1}$ as $0<|e_{2}|\leq \delta$, $e^{k_{j}{\left|e_{2}\right|}^{\alpha_{1}}}>k_{j}{\left|e_{2}\right|}^{\alpha_{1}}>{\left|e_{2}\right|}^{\alpha_{j}-1}$ as $\delta <|e_{2}|\leq 1$ and $e^{k_{j}{\left|e_{2}\right|}^{\alpha_{2}}}>k_{j}{\left|e_{2}\right|}^{\alpha_{2}}>{\left|e_{2}\right|}^{\alpha_{j}-1}$ as $|e_{2}|>1$. Based on this, the gains $\beta_{0j}$ of the traditional ESO and improved nonlinear ESO are set to smaller values, and $\beta_{0j}\lambda_{0j}\left(e_{2}\right)>\beta_{0j}\lambda_{j}\left(e_{2}\right)$. Therefore, compared with a traditional ESO, the improved nonlinear ESO with smaller gains can achieve better estimation performance and suppress noise amplification. In order to reduce the undetermined parameters in the designed observer, we set $\beta_{0j}$ to $\left[\beta_{01},\;\beta_{02},\;\beta_{03}\right]=\left[3\omega_{01},\;3\omega_{01}^{2},\;\omega_{01}^{3}\right],\omega_{01}>1$ based on the parameter setting method proposed in [29]. Moreover, we analyzed the boundedness of the estimation errors of the proposed improved nonlinear extended state observer with the following Theorem 1.

**Theorem** **1.** *For the rotation system Equation (11), the estimation errors of the designed improved nonlinear extended state observer, represented by Equation (16), with $\omega_{0}>1$ are bounded.*

**Proof of Theorem 1.**  The reduced-order state equation of the rotation system with the extended state $x_{4}$ can be rewritten as follows:

(20) $$\left\{\begin{array}{c} {\dot{x}}_{2}=x_{3}\\ {\dot{x}}_{3}=\hat{f}u+x_{4}\\ {\dot{x}}_{4}=\gamma_{1}(x,t) \end{array}\right.$$

Define $\omega_{0}=\omega_{01}\lambda_{01}$, then $\omega_{0}>1$ due to $\omega_{01}>1$. The improved nonlinear extended state observer, represented by Equation (16), can be rewritten as follows:

(21) $$\left\{\begin{array}{c} {\dot{\hat{x}}}_{2}={\hat{x}}_{3}-3\omega_{0}e_{2}\\ {\dot{\hat{x}}}_{3}=\hat{f}u+{\hat{x}}_{4}-3\omega_{0}^{2}e_{2}\\ {\dot{\hat{x}}}_{4}=-\omega_{0}^{3}e_{2}+\gamma_{1}(\hat{x},t) \end{array}\right.$$

Define ${\tilde{x}}_{\tau }={\hat{x}}_{\tau }-x_{\tau },\tau =2,3,4$ as the state estimation error. Based on Equations (20) and (21), the estimation error dynamic of the proposed observer can be expressed as follows:

(22) $$\left\{\begin{array}{c} {\dot{\tilde{x}}}_{2}={\tilde{x}}_{3}-3\omega_{0}{\tilde{x}}_{2}\\ {\dot{\tilde{x}}}_{3}={\tilde{x}}_{4}-3\omega_{0}^{2}{\tilde{x}}_{2}\\ {\dot{\tilde{x}}}_{4}=\gamma_{2}(\hat{x},t)-\gamma_{2}(x,t)-\omega_{0}^{3}{\tilde{x}}_{2} \end{array}\right.$$

Let $\varepsilon_{\tau }\;=\;\frac{{\tilde{x}}_{\tau }}{\omega_{0}^{\tau -2}}$ and $\dot{\varepsilon }={\left[\varepsilon_{2},\varepsilon_{3},\varepsilon_{3}\right]}^{T}$, then one obtains

(23) $$\dot{\varepsilon }\;=\;\omega_{0}A\varepsilon +B\frac{\gamma_{2}(\hat{x},t)-\gamma_{2}(x,t)}{\omega_{0}^{2}}$$

where $A=\left[\begin{array}{ccc} -3 & 1 & 0\\ -3 & 0 & 1\\ -1 & 0 & 0 \end{array}\right]$ is Hurwitz, $B={\left[\begin{array}{ccc} 0 & 0 & 1 \end{array}\right]}^{T}$.

Since *A* is Hurwitz, we can obtain $A^{T}P_{h}+P_{h}A=-I$, where $P_{h}$ is a positive definite matrix and *I* is an identity matrix. Take the following Lyapunov function:

(24) $$V_{0}=\varepsilon^{T}P_{h}\varepsilon$$

Based on Equation (24), one obtains

(25) $${\dot{V}}_{0}=-\omega_{0}{∥\varepsilon ∥}^{2}+2\varepsilon^{T}P_{h}B\frac{\gamma_{2}(\hat{x},t)-\gamma_{2}(x,t)}{\omega_{0}^{2}}$$

Define $\bar{x}={\left[x_{2},x_{3},x_{4}\right]}^{T}$, $\tilde{\bar{x}}=\hat{\bar{x}}-\bar{x}$. We can obtain $\left|\gamma_{2}(\hat{x},t)-\gamma_{2}(x,t)\right|\leq \zeta^{\prime }∥\hat{\bar{x}}-\bar{x}∥$ with $\zeta^{\prime }>0$, then

(26) $$2\varepsilon^{T}P_{h}B\frac{\gamma_{2}(\hat{x},t)-\gamma_{2}(x,t)}{\omega_{0}^{2}}\leq 2\varepsilon^{T}P_{h}B\zeta^{\prime }\frac{∥\varepsilon ∥}{\omega_{0}^{2}}$$

Due to $\frac{∥\hat{\bar{x}}-\bar{x}∥}{\omega_{0}^{2}}=\frac{∥\tilde{\bar{x}}∥}{\omega_{0}^{2}}=\frac{∥\sqrt{\varepsilon_{1}^{2}+\varepsilon_{2}^{2}\omega_{0}^{2}+\varepsilon_{3}^{2}\omega_{0}^{4}}∥}{\omega_{0}^{2}}\leq ∥\varepsilon ∥$ with $\omega_{0}>1$, then

(27) $$2\varepsilon^{T}P_{h}B\frac{\gamma_{2}(\hat{x},t)-\gamma_{2}(x,t)}{\omega_{0}^{2}}\leq \zeta {∥\varepsilon ∥}^{2}$$

where $\zeta =1+{∥P_{h}B\zeta^{\prime }∥}^{2}$. Based on Equations (26) and (27), one obtains

(28) $${\dot{V}}_{0}\leq -\left(\omega_{0}-\zeta \right){∥\varepsilon ∥}^{2}$$

From Equation (28), it can be found that ${\dot{V}}_{0}\leq 0$ as $\omega_{0}>\zeta >1$. Then, the boundedness of the estimation errors of the designed improved nonlinear extended state observer with $\omega_{0}>\zeta >1$ is proven. □

### 3.3. The Sliding-Mode Control Law

In this section, an integral sliding-mode surface is first developed. Then, in order to improve the robustness of the initial state of the system to the sliding-mode surface, we design an improved sliding-mode reaching law. Based on this, the devised sliding-mode control law is presented.

Denote $e=x_{d1}-x_{2}$ as the tracking error. The integral sliding-mode surface for the rotation system is presented as follows:

(29) $$s=e+\int_{0}^{t}edt+\dot{e}$$

In order to improve robustness, the sliding-mode reaching law is usually adopted for sliding-mode control. The commonly-used sliding-mode reaching laws [30,31], such as isokinetic and power reaching laws, are described as follows:

(30) $$\dot{s}=-\tau_{1}\mathrm{sgn}\left(s\right)$$

(31) $$\dot{s}=-\tau_{1}|s{|}^{a}\mathrm{sgn}\left(s\right)$$

where $\tau_{1}>0$, 0<a<1.

Although the reaching laws expressed by Equations (30) and (31) improve the robustness of sliding-mode control, chattering exists due to the use of sign function, leading to the deterioration of the control performance. Due to the non-repeatability of drilling operations, we desire no chattering in rotation speed. In view of this, the following improved sliding-mode reaching law is designed to improve the robustness of the controller and eliminate chattering.

(32) $$\dot{s}=-\tau_{1}e^{|s{|}^{a}}\mathrm{sat}\left(s\right)-\tau_{2}s^{b}$$

with

(33) $$\mathrm{sat}\left(s\right)=\left\{\begin{array}{cc} \mathrm{sgn}(s) & |s|>\phi \\ \frac{s}{\phi } & |s|\leq \phi \end{array}\right.$$

In the above formulas, $\tau_{2}>0$, 0<b<1, 0<ϕ<1. $s^{b}$ is an odd function that depends on *b*. Moreover, a lemma [32] is introduced to prove the fixed-time reachability of the presented improved sliding-mode reaching law in Theorem 2.

**Lemma** **1** ([32]). *Suppose the derivative of a positive definite function $V_{1}$ can be expressed as follows:*

(34) $${\dot{V}}_{1}\leq -\tau_{3}V_{1}^{\chi_{1}}-\tau_{4}V_{1}^{\chi_{2}}$$

*Then the system obeys the following fixed-time convergence.*

(35) $$t\leq \frac{1}{\tau_{3}\left(1-\chi_{1}\right)}+\frac{1}{\tau_{4}\left(\chi_{2}-1\right)}$$

*In the above formulas, $\tau_{3}>0$, $\tau_{4}>0$, $0<\chi_{1}<1$, $\chi_{2}>1$. t is the convergencetime.*

**Theorem** **2.** *The proposed improved sliding-mode reaching law expressed by Equation (32) can make the initial state of the rotation system reach the sliding-mode surface in the following limited time.*

(36) $$t\leq \frac{2\phi }{\tau_{1}2^{\left(1+0.5a\right)}a}+\frac{2}{\tau_{2}2^{0.5(1+b)}\left(1-b\right)}$$

**Proof of Theorem 2.**  Choose the following positive definite function.

(37) $$V_{1}=\frac{1}{2}s^{2}$$

Then, the derivative of the above formula is calculated as follows:

(38) $${\dot{V}}_{1}\;=\;s\dot{s}$$

As |s|>ϕ, we can obtain $e^{|s{|}^{a}}>|s{|}^{a}$, an

(39) $$\begin{array}{cc} {\dot{V}}_{1}=\; & s(-\tau_{1}e^{|s{|}^{a}}\mathrm{sgn}\left(s\right)-\tau_{2}s^{b})\\ =\; & -(\tau_{1}e^{|s{|}^{a}}|s|+\tau_{2}{\left|s\right|}^{1+b})\\ \leq \; & -\tau_{1}|s{|}^{1+a}-\tau_{2}{\left|s\right|}^{1+b} \end{array}$$

As |s|≤ϕ, then

(40) $$\begin{array}{cc} {\dot{V}}_{1}=\; & s(-\tau_{1}e^{|s{|}^{a}}\frac{s}{\phi }-\tau_{2}s^{b})\\ =\; & -(\tau_{1}e^{|s{|}^{a}}\frac{{\left|s\right|}^{2}}{\phi }+\tau_{2}{\left|s\right|}^{1+b})\\ \leq \; & -\frac{\tau_{1}}{\phi }|s{|}^{2+a}-\tau_{2}{\left|s\right|}^{1+b} \end{array}$$

According to Equations (39) and (40), we can obtain ${\dot{V}}_{1}\leq -\frac{\tau_{1}}{\phi }|s{|}^{2+a}-\tau_{2}{\left|s\right|}^{1+b}$, that is ${\dot{V}}_{1}\leq -\frac{\tau_{1}}{\phi }2^{\left(1+0.5a\right)}V_{1}^{\left(1+0.5a\right)}-\tau_{2}2^{0.5(1+b)}V_{1}^{0.5(1+b)}$. Based on Lemma 1, since 1+0.5a>1 and 0<0.5(1+b)<1, the proposed improved sliding-mode reaching law yields the following fixed-time reachability.

(41) $$t\leq \frac{2\phi }{\tau_{1}2^{\left(1+0.5a\right)}a}+\frac{2}{\tau_{2}2^{0.5(1+b)}\left(1-b\right)}$$

□

Moreover, define $e_{4}={\hat{x}}_{4}-x_{4}$ as the estimation error of $x_{4}$; ${\hat{e}}_{4}$ and ${\tilde{e}}_{4}$ as the estimation and estimation error of $e_{4}$ and ${\tilde{e}}_{4}={\hat{e}}_{4}-e_{4}$. ${\hat{e}}_{4}$ is tuned by the adaptation law presented later. Based on the sliding-mode surface and reaching law, the sliding-mode control law, represented by $u_{0}$, is designed as follows:

(42) $$u_{0}=\tau_{1}e^{|s{|}^{a}}\mathrm{sat}\left(s\right)+\tau_{2}s^{b}+\dot{e}+e+{\ddot{x}}_{d1}+{\hat{e}}_{4}$$

### 3.4. The Adaptation Laws

Although the designed improved nonlinear extended state observer has better estimation performance, it will inevitably produce estimation errors. As we know, disturbance estimation error and uncertain gains will reduce the control performance of the system. In order to solve the problem, we designed the following adaptation laws.

(43) $${\dot{\hat{e}}}_{4}=ks$$

(44) $$\dot{\hat{f}}=k\left(1-k_{4}\right)us$$

where k>0, $0<k_{4}<1$.

### 3.5. The Design and Analysis of SMC-INESO

The estimated disturbance is compensated in the sliding-mode control law and the SMC-INESO is constructed as follows.

(45) $$u=\frac{u_{0}-{\hat{x}}_{4}}{k_{4}\hat{f}}$$

The stability of the SMC-INESO-based rotation system is analyzed in the following Theorem 3 according to the Lyapunov theory.

**Theorem** **3.** *For the rotation system Equation (11), the proposed SMC-INESO can guarantee that the tracking error converges to zero.*

**Proof of Theorem 3.**  Select the following Lyapunov function:

(46) $$V=\frac{1}{2}ks^{2}+\frac{1}{2}{\tilde{e}}_{4}^{2}+\frac{1}{2}{\hat{f}}^{2}$$

Based on Equations (29), (45), and (46), one obtains

(47) $$\begin{array}{cc} \dot{V}=\; & ks\dot{s}+{\tilde{e}}_{4}{\dot{\hat{e}}}_{4}+\hat{f}\dot{\hat{f}}\\ =\; & ks\left(\dot{e}+e+\ddot{e}\right)+{\tilde{e}}_{4}{\dot{\hat{e}}}_{4}+\hat{f}\dot{\hat{f}}\\ =\; & ks\left(\dot{e}+e+{\ddot{x}}_{d1}-\hat{f}u-x_{4}\right)+{\tilde{e}}_{4}{\dot{\hat{e}}}_{4}+\hat{f}\dot{\hat{f}}\\ =\; & ks\left[\dot{e}+e+{\ddot{x}}_{d1}-k_{1}\hat{f}u-\left(1-k_{4}\right)\hat{f}u-x_{4}\right]+{\tilde{e}}_{4}{\dot{\hat{e}}}_{4}+\hat{f}\dot{\hat{f}}\\ =\; & ks\left[\dot{e}+e+{\ddot{x}}_{d1}-k_{1}\hat{f}u-x_{4}\right]-\left(1-k_{4}\right)\hat{f}us+{\tilde{e}}_{4}{\dot{\hat{e}}}_{4}+\hat{f}\dot{\hat{f}}\\ =\; & ks\left[\dot{e}+e+{\ddot{x}}_{d1}-u_{0}+{\hat{x}}_{4}-x_{4}\right]-\left(1-k_{4}\right)\hat{f}us+{\tilde{e}}_{4}{\dot{\hat{e}}}_{4}+\hat{f}\dot{\hat{f}}\\ =\; & ks\left[\dot{e}+e+{\ddot{x}}_{d1}-u_{0}+{\hat{e}}_{4}\right]-k{\tilde{e}}_{4}s-\left(1-k_{4}\right)\hat{f}us+{\tilde{e}}_{4}{\dot{\hat{e}}}_{4}+\hat{f}\dot{\hat{f}}\\ =\; & ks\left[\dot{e}+e+{\ddot{x}}_{d1}-u_{0}+{\hat{e}}_{4}+\tau_{1}e^{|s{|}^{a}}\mathrm{sat}\left(s\right)+\tau_{2}s^{b}\right]-ks\left[\tau_{1}e^{|s{|}^{a}}\mathrm{sat}\left(s\right)+\tau_{2}s^{b}\right]\\ \; & -{\tilde{e}}_{4}\left(ks-{\dot{\hat{e}}}_{4}\right)-\hat{f}\left[\left(1-k_{4}\right)us-\dot{\hat{f}}\right] \end{array}$$

Substituting Equations (42)–(44) into the above formula, one obtains

(48) $$\begin{array}{c} \dot{V}=-ks\left[\tau_{1}e^{|s{|}^{a}}\mathrm{sat}\left(s\right)+\tau_{2}s^{b}\right]\leq 0 \end{array}$$

According to Lyapunov stability theory, $\dot{V}\leq 0$ indicates that the SMC-INESO-based rotation system is stable and the tracking error of the system converges to zero. □

The controller proposed in the paper is mainly composed of an improved nonlinear ESO, a sliding-mode control law, and two adaptation laws. Compared with the traditional ESO, the improved nonlinear ESO is slightly more complicated, but has better estimation performance. Though the proposed reaching law has a more complex structure than a traditional sliding-mode control law with an isokinetic reaching law or a power one, it has more robustness. Moreover, the adaptation laws contributes to better tracking performance, but add to the components of SMC-INESO.

**Remark** **1.** *SMC-INESO is composed of a transition process, an improved nonlinear ESO, a sliding-mode control law, and two adaptation laws. There are many key parameters in the above strategies and their settings are critical to control performance. Based on the specific parameter tuning methods in ADRC [27] and SMC [30], we analyzed the roles and settings of the parameters in this paper as follows:*

*(1) In the transition process, there are three parameters to be set, namely r, h, and $h_{0}$. Among them, r is the speed factor, which determines the tracking speed of the system. It can be flexibly set according to the preset rotation speed. h is the integration step, which is usually set to a small constant. $h_{0}$ is set to satisfy $h_{0}>h$, which can eliminate the overshoot phenomenon in the response, thereby suppressing the noise amplification in the differential signal.*

*(2) In improved nonlinear ESO, $\beta_{0j}$ is flexibly set depending on $\omega_{01}>1$. $a_{j}$ is set to $0<a_{3}<a_{2}<a_{1}=1$, based on $a_{j}$; $\alpha_{1}$ and $\alpha_{2}$ are set to $\alpha_{1}<a_{j}-1<\alpha_{2}$. $k_{j}$ is set to satisfy $k_{3}=\frac{3}{2}k_{2}=3k_{1}\geq 3$. The above-mentioned parameter setting method can ensure the fast and accurate estimation performance of the proposed observer. In addition, Theorem 1 also proves the boundedness of the observer’s estimation errors under the above-mentioned parameter settings.*

*(3) In the sliding-mode control law, $\tau_{1}>0$, $\tau_{2}>0$, 0<a<1, 0<b<1 and 0<ϕ<1 are set according to the specific parameter tuning methods in [30] to contribute the good tracking performance of the rotation system.*

*(4) In the adaptation laws, the SMC-INESO tunes the estimated speed of the disturbance estimation error and the uncertain gain estimation according to the settings k>0 and $0<k_{4}<1$, so as to contribute to the fast and good tracking response of the rotation system.*

## 4. Simulation Studies

For the purpose of verifying the effectiveness of the developed controller, five control methods are compared with SMC-INESO. In the SMC-INESO, h=0.002, $h_{0}=0.01$, $\alpha_{1}=-0.75$, $\alpha_{2}=0.5$, $\tau_{1}=0.5$, $\tau_{2}=0.5$, a=0.5, $b=\frac{1}{3}$, $\omega_{01}$ = 6, r=1, k=1, $k_{1}$=1, $k_{2}$ = 2, $k_{3}$ = 4, and $k_{4}$ = 0.8.

### 4.1. Effectiveness Analysis of Proposed Strategies

In this section, five control methods are used as comparison methods to verify the effectiveness of the proposed strategies in the proposed controller, as follows.

(1) SMC-INESO with a linear sliding-mode surface (SMC-INESO-LSS). The linear sliding-mode surface and sliding-mode control law are designed as follows:

(49) $$s=k_{5}e+\dot{e}$$

(50) $$u_{0}=\tau_{1}e^{|s{|}^{a}}\mathrm{sat}\left(s\right)+\tau_{2}s^{b}+k_{5}\dot{e}+{\ddot{x}}_{d1}+{\hat{e}}_{4}$$

where $k_{5}=1$.

(2) SMC-INESO with a power reaching law (SMC-INESO-PRL). The sliding-mode control law is designed as follows:

(51) $$u_{0}=\tau_{1}|s{|}^{a}\mathrm{sgn}\left(s\right)+\dot{e}+e+{\ddot{x}}_{d1}+{\hat{e}}_{4}$$

(3) SMC-INESO with a traditional linear extended state observer (SMC-INESO-LESO) in which $\beta_{01}=100$, $\beta_{02}=600$, and $\beta_{03}=1000$.

(4) SMC-INESO with a traditional nonlinear extended state observer (SMC-INESO-NESO) in which $\beta_{01}=100$, $\beta_{02}=600$, $\beta_{03}=1000$, $\alpha_{1}=1$, $\alpha_{2}=0.75$, and $\alpha_{3}=0.5$.

(5) SMC-INESO without the adaptation laws (SMC-INESO/AL).

**Figure 5:**
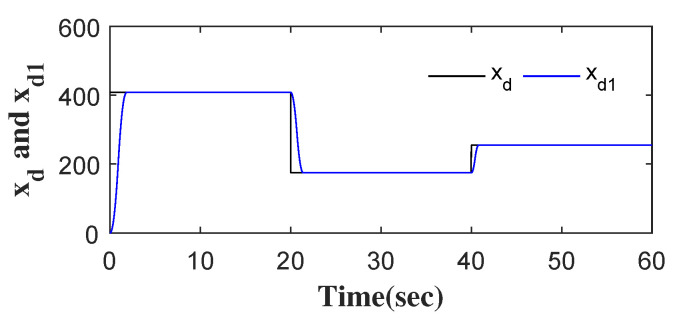
The signal of $x_{d}$ and $x_{d1}$.

**Figure 6:**
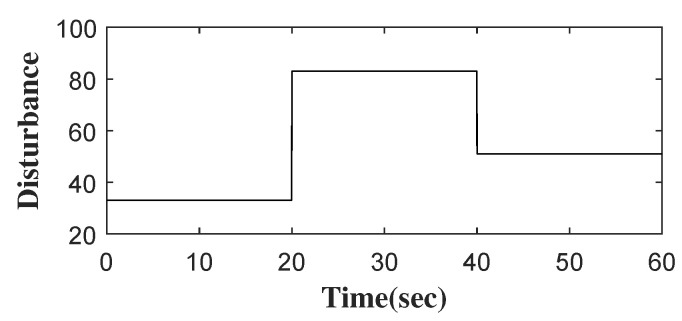
The disturbance.

Considering roadway support in Jincheng Sihe Coal Mine [3], the surrounding rock characteristics of the roadway are listed in Table 1. The rotation speed $x_{d}$ is set according to the characteristics of the surrounding rock. As shown in Figure 5, the step rotation speed $x_{d}$ is converted to a continuous one $x_{d1}$ by the transition process. In the actual roadway support, some elements in the actual rotation system, such as $T_{t}$, may change suddenly and form a sudden disturbance, which is a challenge to the rotation speed control of a hydraulic anchor [3]. The total disturbance, represented by $x_{4}$, is shown in Figure 6.

**Figure 7:**
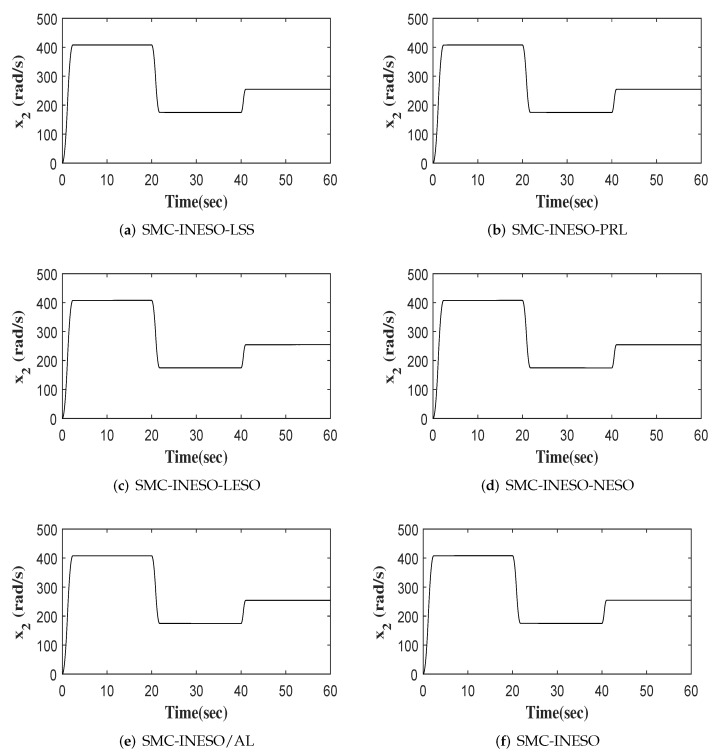
Outputs of six control methods.

**Figure 8:**
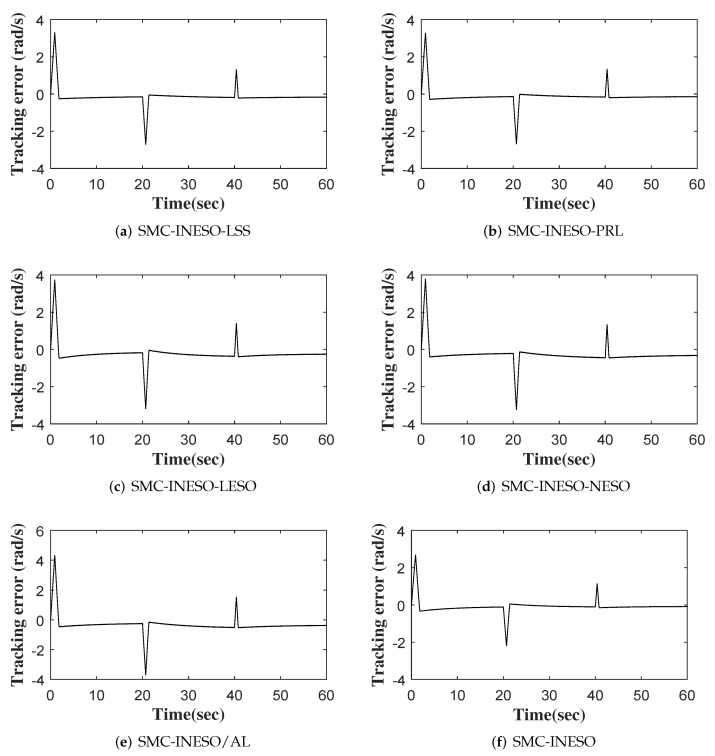
The tracking errors of six control methods.

**Figure 9:**
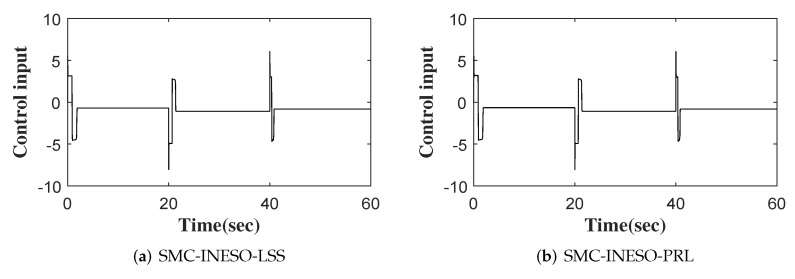

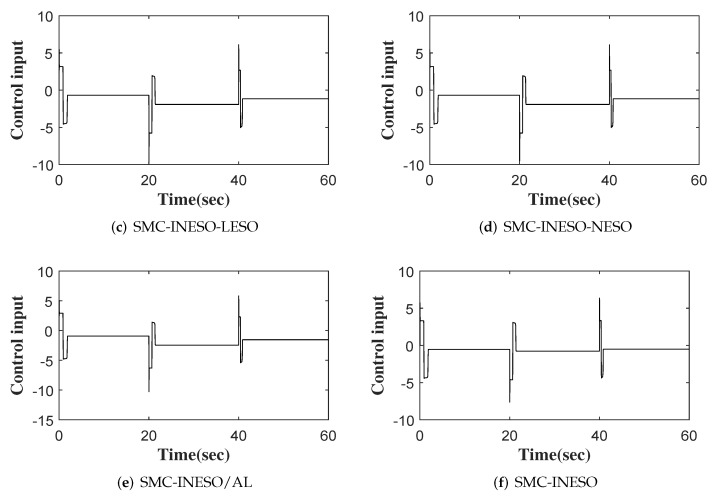
The control inputs of six control methods.

**Figure 10:**
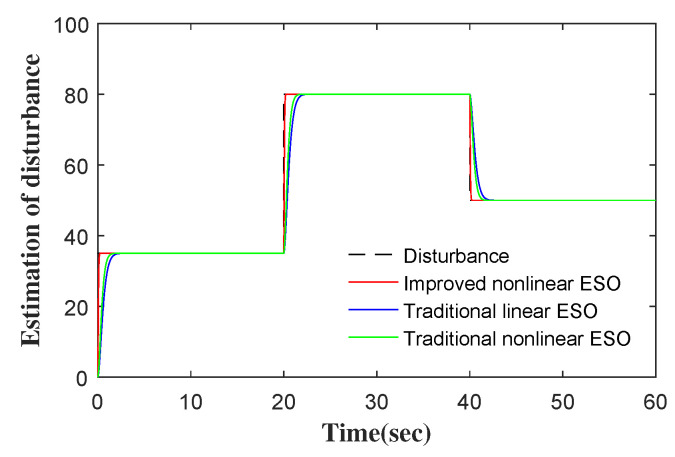
Estimation of disturbances for three ESOs.

The outputs of six comparative control methods are shown in Figure 7. The tracking errors and control inputs of six control methods are shown in Figure 8 and Figure 9. Moreover, as listed in Table 2 and Table 3, eight performance indices in terms of tracking error and control input, i.e., maximum absolute error (MAAE), mean absolute error (MEAE), standard deviation of absolute error (SDAE), integral time absolute error (ITAE), maximum absolute control input (MAACI), mean absolute control input (MEACI), standard deviation of absolute control input (SDACI), and the integral time absolute control input (ITACI), are used to compare the control performance of the six control methods. It can be seen from the experimental results above that the SMC-INESO achieves the smallest average tracking error with the smallest average control input. In addition, by comparing it with the five control methods, it is verified that the strategies proposed in the SMC-INESO, namely the improved nonlinear ESO, the integral sliding-mode surface, the improved sliding-mode reaching law, and the adaptation laws, contribute to the improvement of control performance. In order to verify the estimate performance of the improved nonlinear ESO in the SMC-INESO, traditional linear and nonlinear ESOs are employed for comparison. The estimations of disturbance obtained by the three ESOs are shown in Figure 10. It can be seen from the comparative simulation results that the proposed nonlinear ESO has better estimation accuracy. Therefore, the SMC-INESO with the proposed ESO has stronger anti-disturbance ability.

### 4.2. Effectiveness Analysis of SMC-INESO

In order to verify the effectiveness of SMC-INESO, the following three control methods are employed for comparison.

(1) Adaptive sliding-mode controller (ASMC) [5]

(52) $$u=\frac{u_{0}-\hat{\gamma }}{\hat{f}}$$

(53) $$u_{0}={\ddot{x}}_{d1}+\tau_{21}\left|s{|}^{a_{21}\mathrm{sgn}\left(|s|-1\right)}\mathrm{sat}\left(s\right)+\tau_{22}\right|s{|}^{b_{21}\mathrm{sgn}\left(|s|-1\right)}s$$

(54) $$\hat{\gamma }=-k_{21}s$$

(55) $$\hat{f}=-k_{21}us$$

where $\tau_{21}=\tau_{22}=0.5$, $a_{21}=0.6$, $b_{21}=0.8$, $k_{21}=1$;

(2) Active disturbance rejection control (ADRC) [9]

(56) $$u=\frac{u_{0}-{\hat{x}}_{4}}{f_{0}}$$

(57) $$u_{0}=k_{31}\left(x_{d1}-{\hat{x}}_{2}\right)+k_{32}\left(x_{d2}-{\hat{x}}_{3}\right)$$

where $k_{31}=6$, $k_{32}=8$, ${\hat{x}}_{4}$ is estimated by a traditional linear ESO;

(3) PD control with an improved linear extended state observer (PD-ILESO) [10]

(58) $$u=\frac{u_{0}-{\hat{x}}_{4}}{f_{0}}$$

(59) $$u_{0}=k_{p}e+k_{d}\dot{e}$$

where $k_{p}=10$, $k_{i}=112$, ${\hat{x}}_{4}$ is estimated by an improved linear ESO.

**Figure 11:**
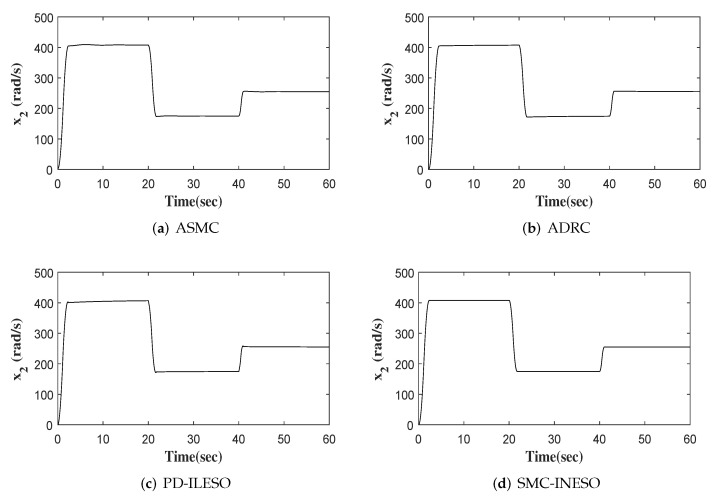
Out responses of four controllers.

**Figure 12:**
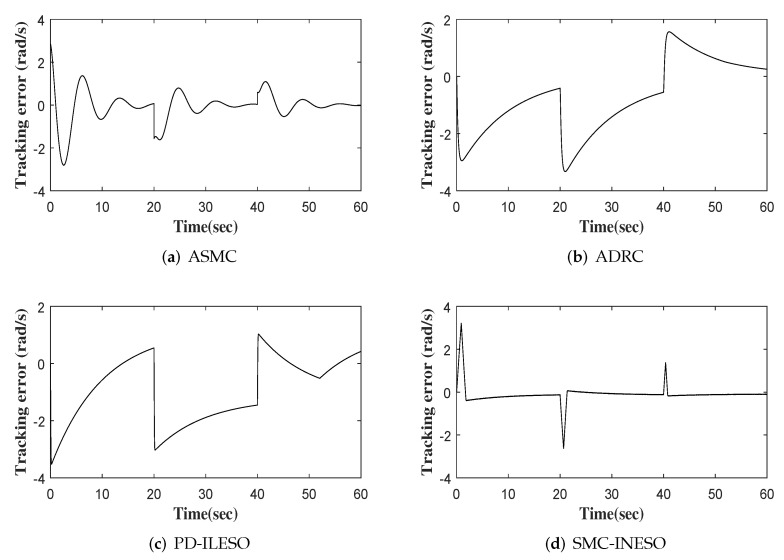
Tracking errors of four controllers.

**Figure 13:**
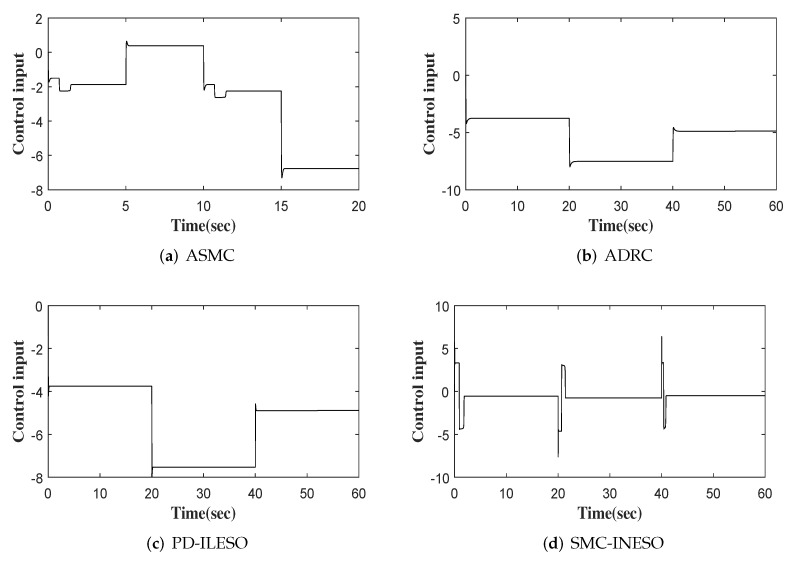
Control inputs of four controllers.

The output, tracking error and control input of four comparative control methods are shown in Figure 11, Figure 12 and Figure 13, respectively. Furthermore, eight performance indices on tracking error and control input are listed in Table 4 and Table 5. We observe from the comparative simulation results that the proposed control method obtains the best indicators in terms of tracking error and control input, meaning that it achieves better control performance.

## 5. Conclusions

In this paper, a SMC-INESO was developed for rotary control of a rotation system with dead zone, disturbances, and uncertain gain. In the SMC-INESO, the improved nonlinear ESO and the sliding-mode control law are integrated. The boundedness of the estimation errors of the improved nonlinear ESO was proven theoretically. The sliding-mode control law was presented based on an integral sliding-mode surface and an improved sliding-mode reaching law. The fixed time reachability of the improved sliding-mode reaching law was proven theoretically. The effectiveness of the SMC-INESO and each strategy were verified through comparative experimental studies. However, the observer designed in this paper is a full-order one, which has a complex structure and contains several undetermined parameters. In view of this, designing a global sliding-mode controller based on a reduced-order ESO for the rotation system will be our future work.

## Figures and Tables

**Table 1:** Characteristics of the rock strata.

Rock Strata	Cumulative Thickness/m	Thickness/m	Pressure/MPa	Strength
Sandy mudstone	10.00	3.67	45.20	5.00
Fine sandstone	6.33	1.10	114.90	12.00
Medium sandstone	4.91	0.55	72.80	8.00
Sandy mudstone	4.36	2.96	31.50	4.00
Coal	1.40	1.40	21.90	3.00

**Table 2:** Performance index of six control methods in terms of *e*.

Control Methods	MAAE	MEAE	SDAE	ITAE
SMC-INESO-LSS	3.2923	0.2468	0.3730	6.3619
SMC-INESO-PRL	3.2823	0.2316	0.3738	6.3721
SMC-INESO-LESO	3.7320	0.3616	0.4122	8.2216
SMC-INESO-NESO	3.7830	0.4108	0.4106	8.4301
SMC-INESO/AL	4.3125	0.4695	0.4693	9.6343
SMC-INESO	2.6771	0.1758	0.3101	5.5562

**Table 3:** Performance index of six control methods in terms of *u*.

Control Methods	MAACI	MEACI	SDACI	ITACI
SMC-INESO-LSS	8.0395	1.0539	0.7789	19.4942
SMC-INESO-PRL	8.0689	1.0423	0.7836	18.7945
SMC-INESO-LESO	9.5250	1.4273	0.8687	19.5950
SMC-INESO-NESO	9.5242	1.4271	0.8687	19.4946
SMC-INESO/AL	10.2945	1.7876	0.9234	23.6942
SMC-INESO	7.6173	0.8134	0.8182	16.6953

**Table 4:** Performance index of six control methods in terms of *e*.

Control Methods	MAAE	MEAE	SDAE	ITAE
ASMC	2.8560	0.4297	0.5196	13.7087
ADRC	3.3266	1.2354	0.7860	27.4323
PD-ILESO	3.5196	1.1281	0.9386	21.1758
SMC-INESO	2.6771	0.1785	0.3101	5.5562

**Table 5:** Performance index of six control methods in terms of *u*.

Control Methods	MAACI	MEACI	SDACI	ITACI
ASMC	7.3089	2.8211	2.3899	56.4090
ADRC	8.0081	5.3890	1.5871	75.0308
PD-ILESO	7.9779	5.3723	1.5824	72.0301
SMC-INESO	7.6173	0.8134	0.8182	16.6953

## Data Availability

Not applicable.
